# Clozapine-Induced Toxic Megacolon: A Rare but Serious Complication

**DOI:** 10.7759/cureus.94228

**Published:** 2025-10-09

**Authors:** Erick A Boldt, Yatin Srinivash Ramesh Babu, Rohan V Rajan, Jeffrey Joppen, Evan Reilly, Sachin Singh

**Affiliations:** 1 College of Osteopathic Medicine, Nova Southeastern University Dr. Kiran C. Patel College of Osteopathic Medicine, Fort Lauderdale, USA; 2 Psychiatry, South Florida Hospital, Miami, USA

**Keywords:** antipsychotic adverse effects, borderline personality, bowel obstruction, clozapine side effects, colonic necrosis, gastrointestinal hypomotility, psychiatry case report, schizoaffective bipolar disorder, schizophrenia, toxic megacolon

## Abstract

Clozapine is a highly effective antipsychotic medication used for treatment-resistant schizophrenia and schizoaffective disorder, with its use most notably associated with the risk of agranulocytosis. As a result, patients on clozapine are routinely screened and monitored for agranulocytosis, but toxic megacolon--a rare and life-threatening gastrointestinal complication--remains an under-recognized adverse effect.

We report the case of a 29-year-old female psychiatric hospital resident with a history of schizoaffective disorder who developed a toxic megacolon while on clozapine therapy. The patient presented with 24 hours of delirium, altered mental status, and a distended, painful abdomen. Despite stabilization efforts, her condition rapidly deteriorated. Emergent decompressive laparotomy revealed a massively dilated colon with solid stool. Postoperatively, she developed abdominal compartment syndrome and underwent a total colectomy for colonic necrosis. Multiple organ failures, including small bowel obstruction, pulmonary emboli, and septic shock, further complicated her clinical course. Despite maximal ICU-level care, she did not survive her condition. This case highlights the need for heightened vigilance and prompt intervention in managing gastrointestinal complications associated with clozapine therapy.

## Introduction

Schizophrenia is a chronic psychiatric disorder characterized by disturbances in thought, perception, and behavior that significantly impair functioning [1]. Although antipsychotic medications form the mainstay of treatment, up to 30% of patients demonstrate inadequate response [2]. For these individuals, clozapine remains the gold-standard therapy for treatment-resistant schizophrenia, with robust evidence supporting its superiority over other agents [3,4].

Despite its efficacy, clozapine’s broad receptor profile is associated with significant adverse effects. Hematologic complications, such as agranulocytosis, are well-known and closely monitored, yet gastrointestinal hypomotility is a less recognized but clinically important side effect [4]. Clozapine’s potent anticholinergic and serotonergic activity slows colonic transit, increasing the risk of severe constipation, pseudo-obstruction, and, in rare cases, toxic megacolon [5,6].

Toxic megacolon, defined as colonic dilation >6 cm with systemic toxicity, is a life-threatening complication most often linked to inflammatory bowel disease or infectious colitis [7]. Its association with clozapine, however, is underreported in the literature and underemphasized in psychiatric training and medical education. Given its high morbidity and mortality, awareness and early recognition are essential. This case highlights a rare and fatal presentation of clozapine-induced toxic megacolon, underscoring the importance of vigilance in patients presenting with abdominal symptoms while on this therapy.

## Case presentation

A 29-year-old female psychiatric hospital resident with schizoaffective disorder, bipolar disorder, and borderline personality disorder presented to the emergency department with a 24-hour history of delirium, altered mental status, and a painful, distended abdomen. The patient was on a regular diet prior to admission. Her psychotropic medication regimen included clozapine 300 mg HS, which is associated with anticholinergic effects, such as constipation and gastrointestinal hypomotility, a known risk factor for bowel obstruction and toxic megacolon. She was also on glycopyrrolate 1 mg TID, a potent anticholinergic, further increasing her risk for gut dysmotility. Additional medications included haloperidol 100 mg IM every four weeks, divalproex sodium 500 mg every morning and 750 mg every night, topiramate 50 mg BID, atorvastatin 20 mg daily, famotidine 20 mg BID, and loratidine PRN.

**Figure 1:**
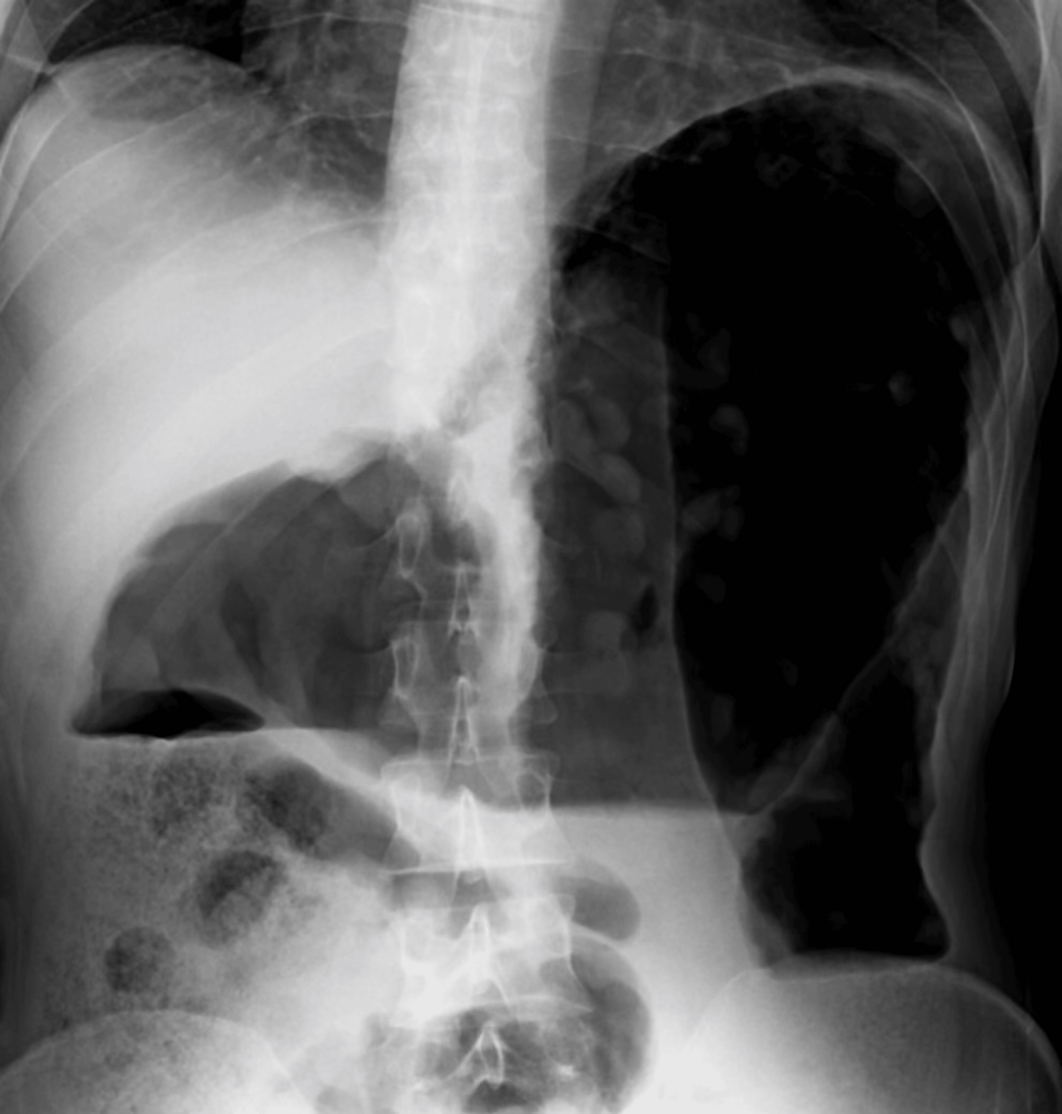
Abdominal radiograph demonstrating marked colonic dilation and loss of haustral markings, consistent with toxic megacolon

Initial evaluation revealed abdominal distension and tenderness, along with systemic signs of toxicity, including hypotension and altered mental status. An abdominal radiograph obtained at the time demonstrated marked colonic dilation and loss of haustral markings, consistent with toxic megacolon (Figure 1). The constellation of symptoms raised concern for an acute abdominal emergency, and she was promptly admitted for further management.

Clinical course

On admission, the patient was confused, hypotensive (BP 85/50 mmHg), and tachypneic (RR 28 breaths per minute). She was intubated for mechanical ventilation and initiated on norepinephrine at 5 mcg/min for blood pressure support. Empiric broad-spectrum antibiotics were started, including vancomycin (15 mg/kg every 12 hours) and piperacillin-tazobactam (4.5 g every 8 hours). An emergent decompressive laparotomy was performed, revealing a massively dilated colon filled with solid stool. Postoperatively, the patient developed abdominal compartment syndrome, requiring close monitoring in the ICU.

On hospital day 2, exploratory laparotomy revealed diffuse colonic necrosis, necessitating a total colectomy. The patient returned to the ICU with an open abdomen. Her postoperative course was complicated by persistent hypotension, managed with vasopressin (0.03 U/min) and norepinephrine titration. Antifungal therapy with micafungin (100 mg daily) and voriconazole (200 mg every 12 hours) was initiated due to suspected fungal superinfection.

Throughout her hospital stay, the patient developed severe hypoxia attributed to bronchial mucus plugging, requiring bronchoscopy with suctioning. Additional complications included small bowel obstruction, thrombosis of the intrahepatic inferior vena cava (IVC) and superior mesenteric vein (SMV), and volume overload managed with a continuous furosemide infusion at 10 mg/hour. A pulmonary embolism was diagnosed on CT imaging, and anticoagulation with a heparin infusion (18 U/kg/h) was started.

Despite maximal supportive care over the next two weeks, including paralytics for hypoxia and escalating antimicrobials, the patient’s condition deteriorated. By hospital day 19, she experienced multiple bradycardic/asystolic arrests. Following discussions with her family, care was transitioned to Do Not Resuscitate (DNR) status. She passed away on hospital day 20. The primary cause of death was bronchopulmonary pneumonia, with her clinical course complicated by large bowel obstruction, sepsis, and multi-organ dysfunction.

## Discussion

Clozapine is regarded as the gold-standard treatment for patients with treatment-resistant schizophrenia and schizoaffective disorders [8]. Despite its noted efficacy, its adverse effect profile often limits its use, necessitating stringent monitoring for hematologic and metabolic complications, a prominent complication being agranulocytosis. This 29-year-old female with schizoaffective disorder, bipolar type, was found to have severe toxic megacolon secondary to her clozapine use, a rare and under-discussed complication.

Although commonly associated with inflammatory bowel disease or infectious colitis, this patient’s presentation implicates a clozapine-induced gastrointestinal hypomotility (CIGH) as the etiology of the severely dilated colon. Toxic megacolon is characterized by an acute colonic distension with severe toxicity and an extremely high risk of perforation. While an uncommon complication, this pathology implicates a severe mortality and morbidity rate, and there is currently a lack of literature related to this disease [9]. Clozapine’s mechanism of action involves anticholinergic and serotonergic effects, which compound to inhibit gastrointestinal smooth muscle contraction and peristalsis. Additionally, clozapine’s antagonism to muscarinic, serotonergic (5-HT3), and dopaminergic receptors contributes to impaired colonic smooth muscle contraction and delayed transit time, which underlies the high prevalence of constipation and predisposes to severe complications such as toxic megacolon [10]. In this case, the concomitant use of glycopyrrolate, another potent anticholinergic, may have compounded the risk of gastrointestinal hypomotility; however, clozapine remains the agent most consistently linked to toxic megacolon in the literature.

The patient’s clinical picture trajectory highlights the crucial challenges in diagnosing clozapine-induced megacolon. The initial vague presentation of delirium and abdominal distension was not immediately identified as secondary to the antipsychotic use, and the patient’s condition further deteriorated due to the lack of urgent treatment. The patient’s further course of multisystem organ failure and repeated invasive interventions underline the significant importance of diagnosing these complications early in the disease progression. This case highlights the need for monitoring routine bowel habits along with the typical hematological surveillance in patients taking clozapine. This also necessitates a multidisciplinary approach between different providers, as patients having symptoms such as abdominal distension, pain, or changes in mental status should raise suspicion for gastrointestinal pathology, prompting urgent imaging and consultation with gastroenterology or surgery. Routine KUBs (kidney, ureter, and bladder imaging) should be performed to observe for any possible obstruction that may be present.

Comparison with previously published reports shows that life-threatening CIGH is a documented but rare phenomenon. For example, Palmer et al. (2008) analyzed 102 life-threatening CIGH cases, reporting a mortality rate of ~27.5% and frequent need for bowel resection [9]. In the UK, pharmacovigilance data (Handley et al.) described 527 harmful CIGH reports between 1992 and 2017, with 172 deaths and many non-fatal cases also undergoing major surgical interventions [11]. Single case reports similarly describe toxic megacolon, bowel ischemia/infarction, and fatal outcomes [12]. Our case aligns with these in terms of severity, outcome, and contributing risk factors.

Most importantly, prophylactic gastrointestinal measures should be emphasized moving forward to prevent disease progression. Previous studies have indicated that utilizing laxatives significantly decreases colonic transit time in patients taking clozapine and reduces medication hypomotility [13]. While clozapine remains the first line for treatment-resistant schizophrenic and schizoaffective disorders, awareness of its full adverse effect profile is crucial for optimizing patient care. Future studies on toxic megacolon and other uncommon complications are vital in refining treatment guidelines and monitoring. Applying standard causality assessment frameworks, this case would be categorized as a probable adverse drug reaction under the Naranjo and WHO-UMC (Uppsala Monitoring Centre) criteria, and the severity would be considered severe/fatal given the need for surgical intervention and the fatal outcome [14,15].

## Conclusions

This case highlights the severe and rare complication of toxic megacolon in a 29-year-old female with treatment-resistant schizoaffective disorder, bipolar type, and borderline personality disorder treated with clozapine. While clozapine’s efficacy in managing refractory psychosis is well-documented, its potential to cause life-threatening gastrointestinal side effects, such as toxic megacolon, remains under-recognized. The patient’s unique presentation and subsequent poor outcome underscore the importance of monitoring for gastrointestinal symptoms in patients on clozapine, particularly those with risk factors such as gastritis or prior gastrointestinal pathology. Early recognition and timely intervention are critical in improving prognosis and preventing fatal outcomes in similar cases.
